# Motives and Commitment to Sport in Amateurs during Confinement: A Segmentation Study

**DOI:** 10.3390/ijerph17207398

**Published:** 2020-10-11

**Authors:** Salvador Angosto, Rosendo Berengüí, José Miguel Vegara-Ferri, José María López-Gullón

**Affiliations:** 1 Department of Physical Activity and Sport, Faculty of Sport Sciences, University of Murcia, 30720 Santiago de la Ribera, Spain; salvador.a.s@um.es (S.A.); josemiguel.vegara@um.es (J.M.V.-F.); 2 Faculty of Social Sciences and Communication, Catholic University of Murcia (UCAM), 30107 Guadalupe, Spain

**Keywords:** motivation, commitment to sport, cluster, amateur athletes, physical activity, sport practise

## Abstract

The current COVID-19 pandemic has paralysed whole countries, which have had to confine their entire population and this changed people’s lives worldwide. The aim of this study is to identify the reasons for and the level of commitment to physical activity among the Spanish population during confinement and the return to the “new normal”. A sample of 1025 amateurs, 534 males and 491 females with an average age of 35 years old were interviewed using an online survey that collected their motives for practising sport and their commitment to physical activity. A cluster analysis combining hierarchical and non-hierarchical methods was performed, identifying three groups of amateurs: High Commitment (*n* = 650), Moderate Commitment (*n* = 324), and Low Commitment (*n* = 81). The main motives shown by the different groups were psychological motives related to an improved or managed general or emotional well-being. Regarding commitment, all the groups showed higher scores in enthusiasm for physical activity than affliction from sport. The variables referring to gender, educational level and sports habits showed differences that enabled the identification of the different groups. These findings highlight the importance of conducting segmentation studies that provide specific population profiles to improve the action strategies of governments and specialists.

## 1. Introduction

The current society is in the middle of a pandemic. COVID-19 is challenging economies and health systems worldwide. Many governments have been forced to carry out home-based confinement as a preventive measure against the spread of the virus [1]. Spain has been one of the most affected countries in the world, forcing the government to decree a state of emergency on 14 March 2020, imposing an obligatory lockdown on the entire Spanish population [2]. Subsequently, on 2 May it established time zones (morning and afternoon) divided into age groups so that the population could engage in outdoor physical activity [3].

The most commonly used strategies to reduce the spread of COVID-19 include restricting travel by closing borders, suppressing mass gatherings, or distancing people socially [4,5]. Consequently, physical activity was restricted to the entire population, leading to the forced closure of sports facilities and recreational areas such as gyms, swimming pools or playgrounds, as well as the cancellation of amateur sports events [6,7].

Despite the current situation, sports participation among the general population has been increasing in recent years due to a greater awareness of the effects that physical activity has on health and the increase of options to be able to do it [8]. In Spain, data from surveys on sports habits have shown an increase in participation of 13.5% between 2010 and 2015 [9]. According to the Global Observatory for Physical Activity [10], Spain has a prevalence of sport participation in males of 73% and in females of 66%, being similar participation of other countries such as the United States (males: 75%; females: 61%) or Japan (males: 69%; females: 64%). The United Kingdom has a slightly lower participation (males: 67%; females: 55%), while in Australia the percentages are lower (males: 45%; females: 42%). In the sports event context, the population’s participation in marathons and other resistance events increased worldwide by 57.8% in recent years [11]. Approximately 50 million Europeans are running regularly [12]. However, a recent study indicates that the Spanish population had a significant decrease in the frequency and intensity of physical activity during confinement and males continue to be more active than females [13].

The motives for practising sport have traditionally been one of the main priorities of sports managers and researchers, as they have to understand the reasons that lead participants to practise sport [14]. Motivation is one of the most important factors in achieving adherence to physical activity [15]. Motivation studies in both sports (i.e., basketball, swimming, etc.) and exercise (i.e., running, cycling, etc.), are often focused on assessing the degree to which individuals report certain motivational qualities [16]. Dosil [17] defines sports motivation as “a psychological factor that encourages the individual to perform, orientate, maintain and/or withdraw from physical-sports activities” (p. 129).

In the sport context, there are several studies in the literature that evaluate motivation in the child population [18,19], adult athletes [20,21,22,23], and recreational or amateur exercisers [24,25,26]. However, no studies have been found that evaluate the motivation of the population during this pandemic period, the psychological aspects being especially relevant since previous studies during periods of confinement have shown the existence of negative impacts, such as increased stress and depression caused by social distancing [27]. Previous studies have indicated that no differences have been observed between the motivations of sportspersons and exercisers, or between individual and team sports athletes [28,29].

Motives in sport are the reasons for this behaviour. They determine the strength, direction, and resistance that the athlete brings to an activity or a result [30]. The concept of motive, although it is closely related to and included in the phenomenon of motivation, refers to the individual’s disposal to perform some action, and to the reasons, with a certain consistency that lead a person to do something [31]. Physical activity behaviour is not usually stimulated by a single reason, rather it would be the sum of several reasons to start the practice [32]. There are many motives, but in adult populations five motives are usually the most cited for engaging in physical activity: (i) for health and fitness; (ii) for the improvement of physical appearance; (iii) for enjoyment; (iv) for socialisation; and (v) for the psychological benefits it brings [32]. Based on the large number of possible motives of adults, different studies have tried to reduce the information and establish categories of motives. Based on the review of Firth et al. [33] three main factors are proposed, which are analysed in this study: (a) Physical: physical health; fitness; strength; weight loss. (b) Psychological: well-being; enjoyment; reduce distress; mood; self-esteem. (c) Socio-ecological: socialising; health professional advice; routine.

Also, the main theories of motivation in physical activity and sport have also explained the value of motives and why people start, maintain, and stop these activities. For example, the two most developed motivational theories in recent years are, on the one hand, the Self-Determination Theory (SDT) [34], which states that motivation and certain behaviours are influenced by certain basic psychological needs, which in this sense can constitute significant motives. On the other hand, the Achievement Goal Theory [35], which assesses goal-directed behaviour, focusing on how individuals approach, engage in, and respond to achievement activities, as well as the motives for engaging in such behaviour. However, there is no specific theory applied in measuring motivation in populations with mental or mood disorders, as is the context in which the review of Firth et al. [33]. This approach is more suitable in an exceptional situation of confinement and social isolation where the majority of the population was not prepared, can induce different problems such as anxiety or depression in the Spanish population [36].

A concept closely associated with motivation is commitment. Commitment is a key aspect in understanding why an athlete is motivated to participate in sport [37]. Vallerand and Young [28] studied the relationships between motivation and sport commitment, finding that different motivations such as enjoyment, stress relief or personal challenges are predictors of commitment. Commitment is a psychological construct that represents the desire and resolve to continue practising physical activity and sport [38]. It is also considered to be a dynamic and psychological state that can vary over time and its relationship with the context, and a commitment to physical activity is necessary for personal health [39].

Positive commitment factors highlight the intrinsic enjoyment of the activity, opportunities to participate successfully and personal investments of time, money, and experience in sport, while negative factors, such as alternatives for successful participation in other attractive sports, lead to decreased commitment [40]. The Sport Commitment Model (SCM), proposed by Scanlan, Chow, Sousa, Scanlan, and Knifsend [41], confirms a series of determinants that represent seven possible sources of sport commitment. The most important are sport enjoyment (the positive affective response to a sport experience that reflects generalised feelings of joy) and valuable opportunities (important opportunities that are only present through continued involvement in a sport). The model also proposes other priorities (alternatives that conflict with continued sport participation); personal investment (personal resources that the athletes put into their sport); social constraints (social expectations that create perceptions of an obligation to remain in a sport); social support (informal, emotional and instrumental); and a desire to shine (including mastery achievement and social achievement) [41].

The segmentation of the population is very useful for both researchers and sports professionals as it enables the differentiation of the different profiles of the amateurs, which allows the establishment of strategies more adapted to the characteristics of each population. Several previous studies have segmented according to the motivation profile of young and adult populations [42,43,44], as well as the profile according to the level of commitment [45,46,47]. It is, therefore, important to be able to assess the motivational profiles and the level of commitment of the population in a pandemic phase, during the final phase of containment and the beginning of the reduction of measures towards the “new normality”. The aim of this study is to assess the motives and commitment of the population to physical activity during the phase of confinement and return to the “new normality”, identifying the latent factors in the motives and commitment. Finally, to compare the socio-demographic and motivational profiles according to the level of commitment to physical activity of the practitioner during the COVID-19 pandemic.

## 2. Materials and Methods

### 2.1. Sample

The study population was considered to be the people who carry out physical activity during their free and leisure time, not receiving any remuneration for the performance of this physical activity, being amateurs. The sample was made up of 1025 people. 52.1% were males and 47.9% were female, with and average age of 35.34 ± 14.2 years old. Table 1 shows the sample’s socio-demographic characteristics. The inclusion/exclusion criteria for the selection of the sample, in addition to being amateur practitioners as described above, had to meet the following requirements: (i) be over 18 years old; (ii) not reside outside the region under study; (iii) a correct response to the control question during the completion of the questionnaire. A total of nine subjects were discarded because they did not meet any of these criteria.

### 2.2. Instrument

The instrument used was a questionnaire structured in three sections. The first section, based on the specific context of the data collection period in which the subjects were in a period of confinement or were returning to the “new normality”, selected the motives that best fit these circumstances. The ad hoc scale of motives for the practice of physical activity was made up of ten items, which were extracted based on the systematic review carried out by Firth et al. [33]. These authors classified the different motives into three different factors (Physical health, Psychological, and Socio-ecological), selecting the statement with the highest response rate of all the studies analysed for each specific motive. The motives selected were related to general health, fitness/energy, general well-being, reducing stress, sleep [48], strength, enjoyment, self-confidence [49], and daily routine [50]. The reliability showed an alpha of Cronbach index of 0.839.

The second section adapted the scale proposed by Parra-Camacho, Alonso, and González-Serrano [46], modifying the terms related to running for sport in general. This scale was composed of 11 items divided into two factors: (i) enthusiasm for the sport (six items); and (ii) affliction with the sport (five items). The reliability showed an alpha of Cronbach index of 0.891. These first and second sections had a five-point Likert response scale (1 = Totally disagree, 2 = Disagree, 3 = Neither agree nor disagree, 4 = Agree, and 5 = Totally agree).

Finally, a third section was composed of the socio-demographic variables, asking about gender, age, education level, marital status and occupation, and some questions related to sport habits (days of weekly sport practice, hours of weekly sport practice, type of activity and years of experience).

### 2.3. Procedure

The procedure carried out in this study consisted of the elaboration of an online questionnaire, using the university’s tool “Surveys”. The sample used was non-probabilistic for convenience, according to the sample’s degree of accessibility. The online survey was distributed among the members of the university itself (researchers and students), specialised forums and its publication in digital media press (local newspapers). The period in which the survey was open was between 24 April and 2 June 2020. This period corresponded, as mentioned above, with the last week of confinement of the population to their homes due to the pandemic and the beginning of the “new normality” with the establishment of time zones for outdoor physical activity according to the age of the population. The population between 18 and 70 years of age had a time slot in the morning from 06:00 to 10:00 and another in the afternoon from 20:00 to 23:00. The survey was totally anonymous. To conduct this study, it was not necessary to have the authorisation of the Bioethics Committee of the local university since, according to the regulations, as it is research that consists of a documentary analysis, a comparative study of the literature (regulations, textbooks, materials, etc.), or involves another type of study in which there is no intervention or contact with human beings (no data are collected from any subject), it is not necessary to request this report.

### 2.4. Data Analysis

Data analysis was performed using the statistical program SPSS v.22.0 (IBM, Armonk, NY, USA). Descriptive statistics were calculated for quantitative variables (mean and standard deviation) and qualitative variables (frequency and percentages). Because the scale of motives was made ad hoc and the scale of commitment to physical activity was adapted from running to sport in general, it was decided to do an Exploratory Factorial Analysis (EFA) with the aim of exploring the possible structure of the underlying factors of the selected motives and commitment [51]. Subsequently, a Confirmatory Factor Analysis (CFA) was sought to verify the factor structure observed in the EFA and the possible relationship between the latent variables [52]. The CFA was calculated using the SPSS AMOS v22.0 statistical programme (IBM, Armonk, NY, USA). This sample division was carried out to avoid the bias of performing the two analyses with the same subjects and to be able to corroborate the structure of each of the variables with a different sample.

To calculate the EFA, we considered the following: the correlation matrix, the Kaiser-Meyer-Olkin (KMO) sample adequacy measure and the Bartlett sphericity test to corroborate the adequacy of the analysis. The EFA was performed by the maximum likelihood method, using the “Oblimin” factor rotation method to motives and “Varimax” rotation to commitment, placing the cross-loading cut-off point at 0.45 [53]. CFA was applied using the maximum likelihood method. To measure the fit of the factors, this study evaluated the model using chi-square statistics, the chi-square ratio and degrees of freedom (*χ*^2^/*df*), the comparative fit index (CFI), the goodness-of-fit index (GFI), the parsimonious normalised fit index (PNFI), the approximation mean square error (RMSEA), and the root-mean-square residue (RMR). Bollen [54] states that an *χ*^2^/*df* ratio with values between 2.0 and 3.0 is considered a suitable fit, as are values even up to 5. Values of CFI above 0.95 and PNFI above 0.6 [55], GFI values above 0.90 [56], and values of RMSEA and RMR below 0.08 would indicate an acceptable model fit [57]. Finally, the reliability of the scale was calculated using Cronbach’s alpha index (C-α), the Composite reliability (CR) and the Analysis of Variance Extracted (AVE). The CR and AVE were estimated to ensure reliability through the results of the CFA, following the indications of Hair, Black, Balin, and Anderson [58].

After checking the variables’ psychometric properties, a cluster analysis was carried out to identify possible groups of participants with a similar level of commitment to physical activity, as not everyone has had the same opportunities for physical activity during the period of confinement, and motivation and commitment varies according to each individual’s situation. The cluster analysis took, as a dependent variable, the total commitment level, calculated from the sum of all the items as proposed by Zarauz and Ruiz-Juan [59]. Two methods were combined in order to obtain the cluster solutions, hierarchical and non-hierarchical, with the aim of optimising the results. The cluster analyses were carried out using the guidelines proposed by Romesburg [60]. The hierarchical cluster was analysed taking Ward’s Method as a reference for the grouping process, while for the similarity measures, the squared Euclidean distance was used. Then, a non-hierarchical cluster was done through the K-means method, taking as a reference the centroids of the cluster solutions of the hierarchical method for each period. Once the ideal cluster solution was determined according to the criteria set out by Hair et al. [58], the profiles of the different groups were determined, using all those variables not included in the cluster analysis. Chi-square tests, calculating the value of the Contingency Coefficient (C^2^) to verify the size effect and the intensity of the association between the qualitative variables compared the results through the performance of the ANOVA test for the continuous variables and for the qualitative variables [61]. The significance level was established at a value of *p* ≤ 0.05.

## 3. Results

### 3.1. Descriptive Results

The descriptive results of the variables are shown in Table 2. The motives towards the practice of physical-sports activity of the participants were mainly related to feeling better about oneself (M = 4.51 ± 0.8), maintaining good health (M = 4.50 ± 0.7) and controlling their psychological well-being (M = 4.41 ± 0.8). In contrast, the motives with lower levels were those related to socio-ecological motives, with scores lower than four points. Based on the right scores, the commitment results showed that the participants had better scores in the items related to enthusiasm for sport, highlighting those related to the desire to do physical activity or sport (M = 4.19 ± 1.0) and that the performance of physical activity is pleasant (M = 4.17 ± 0.9). On the other hand, the participants had a greater affliction for forcing themselves to do more physical activity or sport (M = 2.29 ± 1.2) and doing physical activity is a drudgery task (M = 2.13 ± 1.1), while the rest of the items had scored lower than two points.

### 3.2. Exploratory Analysis Factor

The EFA results of motives and commitment (Table 2) showed that the KMO index had an acceptable value in both cases (Motives = 0.898; Commitment = 0.911), while Bartlett’s sphericity test was significant (Motives: *χ*^2^ (66) = 48,073.86; *p* ≤ 0.001; Commitment: *χ*^2^ (55) = 3282.84; *p* ≤ 0.001). The communalities of all items obtained adequate values above 0.3 [62]. The EFA identified that the different motives were distributed into three factors considering an extraction based on an eigenvalue greater than one. The factors were named based on the categories established by the review carried out by Firth et al. [33], and the context of confinement caused by COVID-19: (i) Physical Health motives are those that deal with aspects related to fitness, physical health or strength; (ii) Psychological motives are those that are closely linked to well-being, mood, and reducing distress or enjoyment; (iii) Socio-ecological or functional motives were so named because of routine aspects which are important in confinement, such as sleep or structuring the day.

In 61.39% of the variance with a solution of three factors, 41.84% belonged to Psychological Motives, 10.45% to Physical Health Motives, and 9.11% to Socio-ecological Motives. The internal structure of the items in the scale showed adequate factor loadings, ranging from 0.576 in the item related to sleep for a maximum of 0.886 in the item relationship with psychological well-being. The internal consistency of each factor in the scale was evaluated by estimating Cronbach’s reliability alpha. According to the criterion of a C-α index equal to or greater than 0.70 [63], the internal consistency was acceptable for Physical Health and Psychological Motives; however, Socio-ecological Motives obtained a value of 0.664, close to 0.700.

On the other hand, the variance explained by the commitment factors was 66.74%, sport enthusiasm being the factor that explained the greatest variance with 50.57%, while affliction explained 16.17%. In relation to the factor loads, all were above 0.4, in accordance with the recommendations of Costello and Osborne [62]. The lowest load was found in the item “I dread the thought of doing sport” with a value of 0.582 and the highest was in the item “Doing sport is vitally important to me” with a factorial weight of 0.845. The reliability values of the C-α index showed very high indices being above 0.8.

### 3.3. Confirmatory Factor Analysis

CFA was used to examine the extent to which the three latent factors extracted from the EFA could be validly replicated. The model was tested using the maximum likelihood parameter estimation method. The results of the CFA of Motives showed that the model was appropriately adjusted (*χ*^2^ (32) = 126.18; *p* ≤ 0.001). The standardised chi-square (*χ*^2^/*df*) obtained a value of 3.943, although the ideal range was between 2.0 and 3.0. Bollen [54] indicates that scores up to 5.0 are acceptable.

The CFI index showed a value of 0.957 while the PNFI had a value of 0.677. These indices were adjusted to the appropriate minimum scores indicated by Hu and Bentler [54]. The GFI also had an acceptable adjustment with a score above 0.90 [56]. Other adjustment indices used were the RMSEA, which had a value of 0.076, and the RMR, with a value of 0.033, with indices below 0.08 being acceptable [56]. According to the CFA of commitment, these results were similar to those obtained in the motive analysis (*χ*^2^ (43) = 229.94; *p* ≤ 0.001). The standardised chi-square showed a score close to 5.0 (*χ*^2^/*df* = 5.35), while all the fit indices had adequate scores (CFI = 0.934; GFI = 0.922; PNFI = 0.720; RMSEA = 0.092; RMR = 0.064).

The CFA load weights of all the items of Motives and Commitment (Table 3) were adequate, the loads complying with the minimum scored recommended (>0.5) and reproducing the initial structure of the EFA. The CR indices of all the factors were above 0.70, in accordance with the recommendations of Hair et al. [58], while the AVE indices are adequate when they are above 0.5. In this case, only the Psychological Motives and Enthusiasm for sport obtained scores up to 0.5. However, it was decided to maintain the other factors because when the reliability of a construct is accepted, slightly lower AVE values could be accepted [64].

### 3.4. Identification and Description of the Clusters

The cluster analysis was carried out with the aim of identifying the participants of the study, according to their level of commitment to physical activity during confinement and the beginning of the “new normality”, using the methodology proposed by Hair et al. [58]. Firstly, the hierarchical cluster analysis (Ward’s Method) was carried out in order to observe the differences in the agglomeration coefficients and their increases between clusters two and three, three and four, and four and five. Subsequently, a non-hierarchical K-means cluster analysis was performed using the solutions of the initial two, three and four cluster centres. It is important to remember that the choice of the ideal cluster depends on different aspects such as the theoretical basis, and the researcher’s common sense or practical judgment [58].

Table 4 shows the centroids of each group of the different items of Motives and Commitment to sport. The results of the ANOVA test confirmed the existence of significant differences in all the variables (*p* ≤ 0.001), except the motive related to maintaining good health that the significant differences were between Cluster 1 with the other two groups. Also, the motive of beneficial for managing psychological well-being and all items of affliction from sport did not show significant differences (*p* > 0.05). The most influential motives in general were those related to enjoyment (*F* = 443.25), followed by those related to improved emotional well-being (*F* = 142.72) and to the motive that exercise makes one feel strong (*F* = 129.17). The Commitment items that had the greatest influence were “Doing sport is pleasant” (*F* = 600.91), “I look forward to doing sport” (*F* = 574.26) and “Doing sport is vitally important to me” (*F* = 497.27).

Cluster 1, named “High Commitment”, was composed by the majority of participants (60.5%) and they were the ones who showed a higher level of commitment to physical activity (M = 49.16 ± 3.3 over a maximum of 55). The main motives of the participants in this group were to maintain good health (M = 4.60 ± 0.6), because sport is fun (M = 4.58 ± 0.6) and to improve their emotional well-being (M = 4.57 ± 0.6). Regarding the items of commitment, those that obtained a higher score were those related to enthusiasm for sport; the most relevant items were the intention to do physical activity (M = 4.68 ± 0.5) and doing sport is pleasant (M = 4.65 ± 0.5), while the affliction from sport factor showed low scores.

Cluster 2 was nominated “Moderately Committed” because the average score of this group at the global commitment level was 28.55 ± 3.5 out of 55 points and was represented by 31.6% of the total sample. The main motives for practising physical-sports activity were to maintain good health (M = 4.37 ± 0.7), feeling well with oneself (M = 4.35 ± 0.8), and to improve one’s emotional state (M = 4.05 ± 0.9). As in Cluster 1, they also generally had higher scores in the enthusiasm for sport factor than in the affliction from sport factor. The items of enthusiasm for the sport had average scores slightly above three points, the best evaluated being looking forward to doing sport (M = 3.73 ± 0.8), less than doing sport is an important part of their day with a score of 2.35 ± 1.1. Concerning the items of affliction from sport, the results were similar to those obtained by the Cluster 1 of high commitment.

Finally, Cluster 3 “Low Commitment” was the smallest group, representing 7.9%, and had a low level of commitment (M = 25.42 ± 4.8). In general, they had the lowest scores on the motives, with the motives best valued being maintained good health (M = 4.26 ± 0.9) and providing benefits for managing psychological well-being (M = 4.09 ± 1.0), being the group with the best score on this motive. This group obtained the lowest scores in enthusiasm for sport among the three groups, highlighting the items “doing sport is pleasant” (M = 2.32 ± 0.9) and they are looking forward to doing sport (M = 2.28 ± 1.0), while affliction from sport scores were similar to values of enthusiasm for sport.

### 3.5. Cluster Profile

The socio-demographic profiles of the different clusters (Table 5) indicated that Cluster 1 “High Commitment” was mainly composed of males (57.7) with an average age of 35.30 ± 13.6 years old, with university studies (68.5%), single (51.3%) and active workers (56.8%). Their sports habits were to perform a physical activity 4.69 days per week, with a total duration of 5.95 ± 3.3 h per week and mainly performed activities related to running, jogging, or cycling (41.0%), followed by individual or collective sports (28.2%). The sports experience was 10.55 years. Cluster 2, “Moderately Committed”, had equal genders, although it was slightly higher in females with 51.2%, aged 35.59 ± 15.1 years, three out four participants had a university or postgraduate studies, half were single and working (51.0%). Their sports habits consisted of practising sports with an average of 4.00 ± 2.1 days/week, 3.95 h/week. This group carried out activities oriented to running or jogging (30.6%) and walking or hiking (19.1%) with a sports experience of 7.53 ± 6.2 years. Finally, Cluster 3 “Low Commitment” was mainly made up of females (77.8%), with an average age of 34.59 ± 15.4 years, 86.5%, with university studies, half were single and were working or studying with 49.4% each. Participants with less commitment indicated that they spent 3.59 ± 2.4 days and 2.63 ± 2.2 h during a week on physical activity. A high percentage went out for a walk or hiking (34.6%) followed by those participants who did fitness activities such as Pilates, yoga, or Zumba (19.8%). The sports experience was 3.83 ± 5.5 years.

## 4. Discussion

The first objective of this study was to assess the motives and commitment of the population to physical activity during the phase of confinement and return to the “new normality”, identifying the latent factors in the motives and commitment to physical activity. On the one hand, the motives were gathered in the same three categories that Firth et al. [33] established in their review study: Physical Health, Psychological and Socio-ecological motives. This categorisation is important, especially in the period in which data have been collected, as during the period of confinement many people have had problems with physical activity due to lack of space or adequate equipment. Although many countries have been flexible in allowing outdoor physical activity with limitations such as once a day and on an individual basis [65], social distancing can have negative impacts such as increased stress and anxiety in the population during a period of confinement [27].

On the other hand, commitment factors results were similar to the study of Parra-Camacho et al. [46], identifying the same two aspects, both positive (Enthusiasm for sport) and negative (Affliction from sport), and obtaining adjustment and reliability indices very close to each other, while in the original Spanish validation, the authors had difficulty in grouping the different items into two factors [59]. Commitment to physical activity is important in the context of a pandemic, as the degree of commitment of the individual is a key element in remaining physically active. Some studies suggest that physical activity can reduce the risk of having acute respiratory distress syndrome, one of the leading causes of death from COVID-19, due to the use of the same respiratory muscles [66,67], as well as allowing an improved immune system response to a respiratory infection [68].

The second objective was to compare the socio-demographic and motivational profiles according to the level of commitment to physical activity of the practitioner during the COVID-19 pandemic. The descriptive results of the main motives referred to by the participants of this study were in line with the social context in which they were involved of confinement and return to the “new normal” during the pandemic, with the highest scores being for psychological motives. These results may be due a high percentage of people tending to engage in physical activity or sport as a means of disconnecting from their daily family or work routine. These results coincided with those shown by the survey of sports habits in Spain [69], where being in a fit state was the main motive expressed for doing sport followed by enjoyment. Several studies on the Latin population have highlighted that being fit has been the main motive for practising sports among adolescents [70], while in the other studies enjoyment and leisure were the main motives for practising sports [71,72].

A few studies which analysed the university population participating in different fitness activities valued the reasons for health and skill development more [73,74,75]. Moreover, these results coincided with other studies carried out in the context of runners. A study, which observed that internationally successful athletes had a competition-oriented motivation, while unsuccessful athletes were oriented towards maintaining good health [21]. Several studies found that the most frequent motives for participating in the event were to maintain a good physical condition or the achievement of personal goals and their personal well-being [24,76,77,78]. Another study of participants in running events observed that enjoyment was one of the most important motives people have to engage in active leisure activities [79]. Furthermore, Waśkiewicz, Nikolaidis, Chalabaev, Rosemann, and Knechtle [80] noted that the most important motives for ultra-runners were the promotion of social relations, health and finding meaning in their lives.

These results were related to previous studies in the population with mental problems. In general, psychological motives scored higher, followed by physical health motives and finally socio-ecological motives [48,49,50,81]. Fraser et al. [48] and Sylvia et al. [50] had as their main motives maintaining good health and physical activity benefiting psychological well-being management, while the highest rated item in this study was feeling better about oneself as did Gorczynski et al. [81].

Motives of enjoyment, stress relief and social affiliation are predictors of engagement in athletes and exercisers [28], while enjoyment is the strongest predictor of functional engagement in master-class swimmers, a similar population to this study [82,83]. The results of the commitment generally showed that the enthusiasm from sport scored higher than affliction for sport. This suggests that the amateurs in this context feel that physical activity is pleasure in the practice itself and the taste for the sport, rather than being an obligation. Research related to exercise and sport suggests a commitment to a two-dimensional construct by reflecting functionality and mandatory forms of engagement [83,84]. Wilson et al. [84] distinguished that functional commitment encompasses all behaviours that people perform at will, by attraction, because of what they want, and predicts a greater participation in physical activity. In addition, Ogles [85] determined that runners with functional commitment were more supportive of fitness motives as their primary reasons for training, while runners with greater obligatory commitment showed better scores on the more extrinsic motives related to achievement and recognition. Also, from the SCM, the sport enjoyment was considered basic, include master’s athletes [41,86,87,88], and commitment was also determined by opportunities for participation and personal investment [40].

Regarding commitment, the results show how the motives most valued by high and moderate commitment groups follow the line of the main theoretical commitment models. The participants primarily value the enjoyment and opportunities that physical activities provide to maintain good physical and mental health. As in the present study, the approaches of Scalan et al. [41] (enthusiastic and constrained commitment), and Wilson et al. [84] (functional and obligatory commitment) proposes two types of commitment. The two cases, both enthusiastic and functional commitments, refer to the positive aspects of commitment and involve voluntary elements of an individual’s commitment decision (“want to”), the enjoyment of the activity being the main source of commitment. Several studies indicate a fundamental role of enthusiastic commitment to sports practice and the enthusiasm and satisfaction with that experience in different sports populations [39,41,45,46,86,89].

Based on what Williams presented [39], the results of this study are significantly influenced by the context in which the research is being carried out, in the COVID-19 period. The commitment to sport of many individuals increases as a measure of enjoyment, opposed to an increase in free time when they stop all social activities and cannot go outdoors. For example, Garcia-Tascon et al. [13] observed a significant increase in the number of females who did not practice physical activity before confinement and began to do so during the pandemic, while females have also adapted better to the circumstances and digital changes than males. This state of engagement may change with the “new normality” as prevention measures are scaled down and social activities are allowed to comply with protocols.

The cluster analysis identified three groups of participants according to the level of general commitment. The name of the groups according to the interpretation of the value of commitment to the practice of physical activity or sport was calculated in line with the sum of all the items as stated by Zarauz et al. [59]. The most important group, representing 60.5%, was called “High commitment”, the intermediate group “Moderate commitment” and the less important group “Low commitment”. A previous study also segmented according to commitment, identifying three groups: Obligatory, Non-commitment, and Enthusiastic [42], while other studies segmented in agreement with motivation by identifying five groups ranging from passive participants to enthusiastic participants [42,90]. The results for the socio-demographic variables showed significant differences between the clusters for the gender and educational level variables. Parra-Camacho et al. [46] also found differences for education level and age, but not according to gender in their sample of amateur runners. Lunas-Aroca and Li-Ping [42] noted significant differences in motives according to gender, age, and marital status. Other studies without a segmentation of the participants observed differences according to gender in runners in Spain [30,59]. The profiles of the three clusters showed a clear difference in gender, but the remaining sociodemographic variables were similar, with the majority with university education, half of the subjects in each group being single, and workers. These results were consistent with other studies except for gender; approximately three out of four subjects were male [46]. The socio-demographic profiles obtained and the differences observed in gender and their degree of commitment can be mainly due to two reasons: (i) a higher proportion of male in the highest commitment group may be because of the fact that females tend to have higher barriers to access physical activity and sport owing to socio-political factors, such as the fact that they traditionally spend more time on domestic work and childcare [91]; and (ii) the differences according to the educational level may be due to two factors, one being the high proportion of students increasing as their level of commitment decreases because of the radical change that they underwent in adapting to virtual teaching, this involved a greater dedication as opposed to spending time on physical activity, and another being that in Spain there is a higher percentage of females who are studying at university and who complete their studies than males [92].

Regarding sports habits, significant differences were observed in all the variables analysed of frequency and duration of sports practice, type of physical activity performed and years of experience. Generally, the “High Commitment” participants spent more days and time practising exercise than the other groups with moderate and low commitment. The results were similar to another study with amateur runners [75]. The type of activity that they performed showed different tendencies according to the level of commitment. In relation to the sociodemographic variables, except age and gender, the results coincided with other studies with recreational runners [93]. In summary, in the sport context, customer segmentation tends to show very heterogeneous profiles [94].

The motivational profile of each group established significant differences in all the motives individually, except the motive of improving the management of psychological well-being. According to their level of commitment to physical activity, the participants also reported differences between the main motives that they had for practising sports. Compared to previous studies that did not segment participants, some studies indicate that males who run tend to focus more on achievement motivations, although not in this case, since they had a high score on psychological factor motives. However, females tend to have a more intrinsic motivation focused on self-esteem, psychological goals, or health [95,96,97]. Another study also reported that males had a clear ego-oriented motivation, while females were motivated by motives about their appearance or physical condition [98]. According to age, several studies found that the main motives for practising sports among people aged between 30 and 55 years old were mood management, skill development, medical requirements, support network, and enjoyment [99,100,101]. In turn, another study indicated that younger adults had a higher intrinsic motivation than middle-aged adults [102]. Several studies indicate that runners who were involved in this practice for between 5 and 10 years do so primarily because of their concern about their weight and their search for social relationships, while those who have been practising this sport for more than 10 years were motivated by concerns about their health, personal goals, the meaning of life, and self-esteem [25,84,103].

Finally, the commitment profile of each group showed that the high and moderate commitment groups scored higher on enthusiasm for the sport than an affliction from sport, and the low commitment group had very similar scores between these two factors. The high commitment group had scores above four points, while the moderate committed group had neutral values close to three points. Both groups highlighted aspects such as the participants looking forward to doing sport and doing sport being pleasant. The affliction from sport analysis showed no significant differences between either group, with similar results, and scores close to two points. These results are consistent with those obtained by Parra-Camacho et al. [46] who observed that the groups with the highest level of commitment had higher scores on enthusiasm for sport relative to affliction from sport. Williams [39] believes that creating environments in which individuals enjoy themselves will lead to greater commitment, becoming a means to better understand the motivation that drives people to practise sports and its continuation [104].

This study has several limitations. Firstly, there is the context in which the data collection was carried out, which can justify the results obtained. The data collection took place during the transition from confinement to post-confinement in Spain, so the pandemic situation may have conditioned the results. Therefore, they should be considered with caution compared to those obtained in previous studies, despite a normal situation being clearly indicated. The current state of emergency has entirely conditioned the practice of sport worldwide, and the main reasons for this may be related to psychological factors and the maintenance of health, as well as, with respect to commitment, the scores being higher in enthusiasm for sport than affliction from sport after the confinement. A final limitation was that the dissemination of the survey in a university context has resulted in a high proportion of the sample being young students.

Consequently, according to these limitations, future studies must contemplate contexts in which the current “new normality” situation is more similar to that before the pandemic in order to observe the differences in the motives and commitment of the amateur population. It is also necessary to carry out a greater number of studies contemplating the general population and not in specific contexts such as runners, who have been widely studied. It would also be interesting to address a more general and less specific population than students, as well as in other countries and contexts. Other studies could also approach the motives and commitment in a common way, segmenting the population to compare the results with other different contexts or analysing their relation to addiction to exercise, especially in the subjects with a high level of commitment.

### Practical Implications

This study allows us to find out the profiles of the motives and commitment of the general population that practises physical activity and sport as amateurs. The literature review showed a lack of studies that evaluate motivation and commitment in the amateur population, and there is also a deficit of studies that perform a segmentation of the participants according to their level of commitment. The findings of this study are especially relevant for government authorities, sports managers and sports groups or other specialists such as psychologists or nutritionists. Understanding the profile of reasons that motivate the amateur population to engage in physical activity and its perspective focused on health and psychological aspects is very important when developing new sports proposals that allow greater adherence to physical activity in this population group. Sports amateurs are more widely represented in the different strata of society than professional athletes and females. It is necessary to take into account the socio-demographic factors and sports habits of amateurs so that sports specialists, psychologists, nutritionists, and leaders know how to adapt to the demand of specific needs and for the population to meet the minimum recommendations for weekly physical activity and a healthy physical and mental state.

Likewise, segmentation studies are especially important in a context such as the current pandemic as they allow the identification of population groups with similar characteristics enabling governments and specialists in sport, health, psychology, etc., to address more specific action strategies with a greater probability of success. Furthermore, sedentary groups and those with a low level of commitment to physical activity are a population with a greater risk of being able to contract the COVID-19 and whose symptoms may be more serious, needing admittance to the healthcare systems which are already overloaded with cases. Also, the segmentation according to the level of commitment is especially useful for local governments and sports teams to develop strategies focused on groups with less commitment to sport with the aim of increasing this commitment and the participation of this group of people in different activities that promote a more active and healthy life.

## 5. Conclusions

The main findings of this study are influenced by the context of the pandemic caused by COVID-19 which forced the population to maintain a period of compulsory confinement in their homes in order to reduce the collapse of the health system and produced a strong negative economic impact. The main reasons observed in the amateur population are related to psychological aspects despite the fact that as observed in the literature, physical reasons are often highlighted.

Furthermore, the segmentation of the participants showed that the large majority had a high commitment to physical activity during this period. This is due to the prevention measures adopted making social activities impossible and more time being made available for physical activity during the hours of confinement. All amateurs show a clear orientation towards enthusiastic commitment despite the background conditions as they find physical activity a means of enjoyment and entertainment.

Finally, socio-demographic profiles show the influence of gender and educational level on the level of commitment of amateurs, with the group with the highest degree of commitment being composed of males with a university education and a higher proportion with high school studies or vocational education. The group with the lowest degree of commitment is made up of females with a higher proportion of people with university studies, showing the possible barriers that females still have to fully access physical activity practice due to having to perform other types of tasks such as housework or childcare during confinement.

## Figures and Tables

**Table 1:** Socio-demographic characteristics of the sample.

Variables	*n*	%
Gender		
Male	534	52.1
Female	491	47.9
Education level		
Elementary Studies	58	5.7
High School/Vocational education	210	20.5
Graduate/Post-graduate	745	72.7
Other	12	1.2
Marital Status		
Single	526	51.3
Married or Cohabiting	451	44.0
Divorced or separated	45	4.4
Widowed	3	0.3
Occupation		
Unemployed	30	2.9
Employed	557	54.3
Retired	29	2.8
Student	409	39.9

**Table 2:** Description of the EFA factors and results.

Variables		M (SD)	Factor Loading	Com.
**Motives**	**Factor 1: Physical Health Motives** (*eigenvalue:1.25; %variance:10.45; C-α:0.705*)			
	To maintain good health.	4.50 (0.7)	0.576	0.359
	To improve my energy levels.	4.13 (0.9)	0.740	0.582
	Exercise makes me feel strong.	4.19 (0.9)	0.687	0.541
	**Factor 2: Psychological Motives** (*eigenvalue:5.02; %variance:41.84; C-α:0.873*)			
	Beneficial for managing psychological well-being.	4.41 (0.8)	0.848	0.797
	I will have fun.	4.09 (1.0)	0.657	0.483
	To improve my emotional well-being.	4.29 (0.9)	0.886	0.793
	To help manage my stress.	4.21 (1.0)	0.785	0.649
	I will feel better about myself.	4.51 (0.8)	0.673	0.471
	**Factor 3: Socio-ecological Motives** (*eigenvalue:1.09; %variance:9.11; C-α:0.664*)			
	It helps me sleep better.	3.95 (1.0)	0.615	0.397
	Exercise helps to structure my day.	3.60 (1.2)	0.723	0.714
**Commitment**	**Factor 1: Enthusiasm for sport** *(eigenvalue:5.56; %variance:50.57; C-α:0.904)*			
	I look forward to doing sport.	4.19 (1.0)	0.707	0.647
	Doing sport is vitally important to me.	3.72 (1.1)	0.845	0.749
	Life is so much richer as a result of doing sport.	3.95 (1.0)	0.824	0.709
	Doing sport is pleasant.	4.17 (0.9)	0.738	0.700
	I would arrange or change my schedule to meet my need to do sport.	3.75 (1.1)	0.707	0.537
	Doing sport is the high point of my day.	3.04 (1.2)	0.743	0.564
	**Factor 2: Affliction from sport** *(eigenvalue:1.78; %variance:16.17; C-α:0.826)*			
	Doing sport is drudgery. (R)	3.87 (1.1)	0.743	0.622
	I do not enjoy doing sport. (R)	4.35 (1.0)	0.741	0.605
	I dread the thought of doing sport. (R)	4.64 (0.9)	0.582	0.341
	I have to force myself to do sport. (R)	3.71 (1.2)	0.639	0.569
	To miss a day doing sport is a sheer relief. (R)	4.52 (0.9)	0.666	0.472

SD: Standar Deviation; Com: Communalities; R: reverse item.

**Table 3:** CFA results.

Variables		Factor Loading	CR	AVE
**Motives**	***Factor 1: Physical Health Motives***		0.70	0.45
	To maintain good health.	0.504		
	To improve my energy levels.	0.692		
	Exercise makes me feel strong.	0.786		
	***Factor 2: Psychological Motives***		0.87	0.58
	Beneficial for managing psychological well-being.	0.806		
	I will have fun.	0.637		
	To improve my emotional well-being.	0.860		
	To help manage my stress.	0.775		
	I will feel better about myself.	0.706		
	***Factor 3: Socio-ecological Motives***		0.64	0.47
	It helps me sleep better.	0.698		
	Exercise helps to structure my day.	0.678		
**Commitment**	***Factor 1: Enthusiasm for sport***		0.90	0.61
	I look forward to doing sport.	0.782		
	Doing sport is vitally important to me.	0.830		
	Life is so much richer as a result of doing sport.	0.817		
	Doing sport is pleasant.	0.816		
	I would arrange or change my schedule to meet my need to do sport.	0.740		
	Doing sport is the high point of my day.	0.677		
	***Factor 2: Affliction from sport***		0.82	0.49
	Doing sport is drudgery.	0.758		
	I do not enjoy doing sport.	0.764		
	I dread the thought of doing sport.	0.599		
	I have to force myself to do sport.	0.727		
	To miss a day doing sport is a sheer relief.	0.623		

CR = Composite Reliability; AVE = Average Variance Extracted.

**Table 4:** Average Motives and Commitment scores in the three clusters.

Variables			High Commitment (*n* = 620)	Moderate Commitment (*n* = 324)	Low Commitment (*n* = 81)	*F (df)*	*p*-Value
			M (SD)	M SD)	M (SD)		
Motivation	Factor 1: Physical Health Motives	To maintain good health.	4.60 (0.6)	4.37 (0.7)	4.26 (0.9)	19.04 (1022)	0.000 ^#^
		To improve my energy levels.	4.35 (0.8)	3.90 (0.9)	3.41 (1.1)	66.44 (1022)	0.000 *
		Exercise makes me feel strong.	4.49 (0.7)	3.87 (0.9)	3.20 (1.2)	129.17 (1022)	0.000 *
	Factor 2: Psychological Motives	Beneficial for managing psychological well-being.	3.88 (1.1)	3.90 (1.1)	4.09 (1.0)	1.30 (1022)	0.272
		I will have fun.	4.58 (0.6)	3.64 (0.9)	2.10 (1.1)	443.25 (1022)	0.000 *
		To improve my emotional well-being.	4.57 (0.6)	4.05 (0.9)	3.12 (1.3)	142.72 (1022)	0.000 *
		To help manage my stress.	4.47 (0.8)	3.99 (1.0)	3.05 (1.3)	107.37 (1022)	0.000 *
		I will feel better about myself.	4.72 (0.5)	4.35 (0.8)	3.56 (1.2)	117.24 (1022)	0.000 *
	Factor 3: Socio-ecological Motives	It helps me sleep better.	4.15 (0.9)	3.76 (1.0)	3.16 (1.3)	43.83 (1022)	0.000 *
		Exercise helps to structure my day.	3.91 (1.0)	3.32 (1.1)	2.40 (1.3)	84.75 (1022)	0.000 *
Commitment	Factor 1: Enthusiasm for sport	I look forward to doing sport.	4.68 (0.5)	3.73 (0.8)	2.28 (1.0)	574.26 (1022)	0.000 *
		Doing sport is vitally important to me.	4.34 (0.7)	3.01 (1.0)	1.91 (0.8)	497.27 (1022)	0.000 *
		Life is so much richer as a result of doing sport.	4.47 (0.7)	3.37 (0.9)	2.27 (1.0)	420.79 (1022)	0.000 *
		Doing sport is pleasant.	4.65 (0.5)	3.71 (0.8)	2.32 (0.9)	600.91 (1022)	0.000 *
		I would arrange or change my schedule to meet my need to do sport.	4.28 (0.8)	3.19 (0.9)	2.01 (1.0)	342.61 (1022)	0.000 *
		Doing sport is the high point of my day.	3.62 (1.0)	2.35 (1.1)	1.40 (0.8)	296.00 (1022)	0.000 *
	Factor 2: Affliction from sport	Doing sport is drudgery.	2.12 (1.1)	2.13 (1.1)	2.21 (1.0)	0.243 (1022)	0.785
		I do not enjoy doing sport.	1.64 (1.1)	1.67 (1.0)	1.62 (1.0)	0.136 (1022)	0.873
		I dread the thought of doing sport.	1.35 (0.9)	1.35 (0.8)	1.40 (0.9)	0.082 (1022)	0.922
		I have to force myself to do sport.	2.27 (1.3)	2.32 (1.2)	2.31 (1.3)	0.202 (022)	0.817
		To miss a day doing sport is a sheer relief.	1.49 (0.8)	1.48 (0.9)	1.33 (0.7)	1.244 (1022)	0.289

SD: Standard Deviation; df: degree of freedom; ^#^ Differences between Cluster 1 and Cluster 2 and Cluster 3; * Differences between all the groups.

**Table 5:** Characteristics of the different groups.

Variables	High Commitment (*n* = 620)	Moderate Commitment (*n* = 324)	Low Commitment (*n* = 81)
	M (SD)	M (SD)	M (SD)
**Age** (*F* = 0.162(1022); *p* = 0.851)	35.30 (13.6)	35.59 (15.1)	34.59 (15.4)
	***n* (%)**	***n* (%)**	***n* (%)**
Gender * (*x*^2^ = 38.3(2); *p* ≤ 0.001; *C*^2^ = 0.190)			
Male	358 (57.7)	158 (48.8)	18 (22.2)
Female	262 (42.3)	166 (51.2)	63 (77.8)
Education level * (*x*^2^ = 38.2(12); *p* ≤ 0.001; *C*^2^ = 0.190)			
Elementary studies	45 (7.5)	13 (4.0)	-
High School/Vocational Education	144 (23.2)	55 (17.0)	11 (13.5)
Graduate/Post-graduate	425 (68.5)	250 (77.2)	70 (86.5)
Other	6 (0.8)	6 (1.8)	-
**Marital Status** (*x*^2^ = 5.76(6); *p* = 0.450; *C*^2^ = 0.075)			
Single	318 (51.3)	166 (51.2)	42 (51.9)
Married or Cohabiting	273 (44.0)	143 (44.1)	32 (43.2)
Divorced or Separated	29 (4.7)	13 (4.0)	3 (3.7)
Widowed	-	2 (0.6)	1 (1.2)
**Occupation** (*x*^2^ = 13.8(10); *p* = 0.182; *C*^2^ = 0.115)			
Unemployed	21 (3.4)	9 (2.8)	-
Employed	352 (56.8)	165 (51.0)	40 (49.4)
Retired	17 (2.7)	11 (3.4)	1 (1.2)
Student	230 (37.1)	139 (42.8)	40 (49.4)
	**M (SD)**	**M (SD)**	**M (SD)**
Days of practice * (*F* = 20.30(1022); *p* ≤ 0.001)	4.69 (1.8)	4.00 (2.1)	3.59 (2.4)
Time of practice (hours/week) * (*F* = 73.59(1022); *p* ≤ 0.001)	5.95 (3.3)	3.95 (2.7)	2.63 (2.2)
Sport Experience * (*F* = 65.56(1022); *p* ≤ 0.001)	10.55 (5.5)	7.53 (6.2)	3.83 (5.5)
	***n* (%)**	***n* (%)**	***n* (%)**
Type of Physical Activity * (*x*^2^ = 193.0(10); *p* ≤ 0.001; *C*^2^ = 0.398)			
Walking or hiking	23 (3.7)	62 (19.1)	28 (34.6)
Running, jogging, biking, etc.	254 (41.0)	99 (30.6)	8 (9.9)
Physical conditioning	111 (17.9)	42 (13.0)	4 (4.9)
Fitness (yoga, pilates, zumba, etc.)	53 (8.5)	49 (15.1)	16 (19.8)
Individual or group sport	175 (28.2)	60 (18.5)	14 (17.3)
Nothing	4 (0.6)	12 (3.7)	11 (13.6)

SD: Standard Deviation; %: Percentage; * Differences *p* ≤ 0.001.
